# Overexpression of Poplar Xylem Sucrose Synthase in Tobacco Leads to a Thickened Cell Wall and Increased Height

**DOI:** 10.1371/journal.pone.0120669

**Published:** 2015-03-25

**Authors:** Zhigang Wei, Zanshuang Qu, Lijie Zhang, Shuanjing Zhao, Zhihong Bi, Xiaohui Ji, Xiaowen Wang, Hairong Wei

**Affiliations:** 1 State Key Laboratory of Tree Genetics and Breeding, Northeast Forestry University, Heilongjiang Harbin, P. R. China; 2 College of Information and Computer Engineering, Northeast Forestry University, Heilongjiang Harbin, P. R. China; 3 School of Forest Resources and Environmental Science, Michigan Technological University, Houghton, Michigan, United States of America; Leibniz-Institute for Vegetable and Ornamental Crops, GERMANY

## Abstract

Sucrose synthase (SuSy) is considered the first key enzyme for secondary growth because it is a highly regulated cytosolic enzyme that catalyzes the reversible conversion of sucrose and UDP into UDP-glucose and fructose. Although SuSy enzymes preferentially functions in the direction of sucrose cleavage at most cellular condition, they also catalyze the synthetic reaction. We isolated a gene that encodes a SuSy from *Populus simonii×Populus nigra* and named it *PsnSuSy2* because it shares high similarity to *SuSy2* in *Populus trichocarpa*. RT-PCR revealed that *PsnSuSy2* was highly expressed in xylem, but lowly expressed in young leaves. To characterize its functions in secondary growth, multiple tobacco overexpression transgenic lines of *PnsSuSy2* were generated via *Agrobacterium*-mediated transformation. The *PsnSuSy2* expression levels and altered wood properties in stem segments from the different transgenic lines were carefully characterized. The results demonstrated that the levels of *PsnSuSy2* enzyme activity, chlorophyll content, total soluble sugars, fructose and glucose increased significantly, while the sucrose level decreased significantly. Consequently, the cellulose content and fiber length increased, whereas the lignin content decreased, suggesting that *PsnSuSy2* plays a significant role in cleaving sucrose into UDP-glucose and fructose to facilitate cellulose biosynthesis and that promotion of cellulose biosynthesis suppresses lignin biosynthesis. Additionally, the noticeable increase in the lodging resistance in transgenic tobacco stem suggested that the cell wall characteristics were altered by *PsnSuSy2* overexpression. Scanning electron microscopy was performed to study the cell wall morphology of stem, and surprisingly, we found that the secondary cell wall was significantly thicker in transgenic tobacco. However, the thickened secondary cell wall did not negatively affect the height of the plants because the *PsnSuSy2*- overexpressing lines grew taller than the wildtype plants. This systematic analysis demonstrated that *PsnSuSy2* plays an important role in cleaving sucrose coupled with cellulose biosynthesis in wood tissue.

## Introduction

Cellulose, the world’s most abundant biopolymer, contains simple linear chains of glucose residues, which are aggregated to form immensely strong microfibrils [1]. These fibers are preferably deposited at the inner surface of the plant cell wall in plant stems, branches and roots, and they play essential roles in transporting water and nutrients as well as providing mechanical support during plant growth and development [2]. After plants are harvested, cellulose can be used in the production of biofuel, timber, forage, fiber and chemical cellulose. Despite its importance, our knowledge of the biosynthesis and regulation of cellulose is still limited. Cellulose is synthesized using UDP-glucose, which is produced in a reversible reaction, sucrose + UDP ⇔ UDP-glucose + Fructose, catalyzed by sucrose synthase (SuSy; EC 2.4.1.13). However, this reaction can be reversed, which entails the more dynamic usage of carbohydrates from photosynthesis. Although sucrose is indispensable for cellulose biosynthesis, the availability of carbohydrates from photosynthesis is generally not a rate-limiting factor in cellulose synthesis for plant growth and maintenance [3]. Rather, the creation of photoassimilation sinks or an increase in the strength of the sinks in cellulose-producing cells can partition a greater portion of the photosynthesis to these cells and boost cellulose production [4–6]. We aimed to explore the regulation of the reversible conversion between sucrose and UPD-glucose + fructose, which is catalyzed by SuSy enzymes. In particular, we investigated the consequences of overexpression of the SuSy enzyme.

It has been shown that SuSy enzymes function in various biological processes, including supplying energy to companion cells for phloem loading [7, 8], providing substrates for starch synthesis [9, 10], determining sink strength [8, 11], and supplying UDP-glucose for the synthesis, integration and thickening of the cell wall [10, 12, 13]. SuSy enzymes exist in two forms, a soluble form (S-SuSy) and a particulate form (P-SuSy). S-SuSy enzymes are mainly present in the cytoplasm of plant cells, and P-SuSy enzymes are usually membrane bound and directly supplies UDP-glucose to the cellulose synthase rosettes for cellulose biosynthesis through metabolic channeling [14–17]. SuSy enzymes regulate the influx of carbon to produce cellulose in the vascular tissues of the stem, which is composed of highly active sink cells [18, 19]. In the sink cells, sucrose synthase is the major sucrolytic enzyme that catalyzes the reversible reaction mentioned above [19]. In maize, phosphorylation of SuSy has been shown to change the enzyme from a membrane-bound form to a soluble form [20, 21], but this phosphorylation also appears to be reversible [22]. SuSy enzymes are also implicated in both the biosynthesis and the mobilization of storage and structural carbohydrates by serving as a catalyst in the metabolism of sucrose. This activity leads to the release of the various precursors for the biosynthesis of other metabolites, such as callose [23], cellulose [16] and mixed linkage(1–3), (1–4)-glucans [24]. It has been demonstrated that suppression of *SuSy* expression in transgenic cotton (*Gossypium hirsutum*) ovules decreases fiber elongation [25] and affects cellulose deposition [26]. However, the fiber length of transgenic tobacco (*Nicotiana tabacum*) with overexpressed *Gossypium hirsutum SuSy* decreases [27]. Moreover, the transgenic cotton with overexpressed potato *SuSy* was reported to have increased fiber yield and fiber length [27, 28]. The transgenic lines of hybrid poplar (*Populus alba×Populus grandidentata*) with up-regulated *Gossypium hirsutum SuSy* have thicker secondary cell wall and greater wood density [29]. Investigations of gene expression patterns in poplar indicate that *SuSy* is associated with cellulose synthesis [30–32]. For example, *PtSuSy1* and *PtSuSy2* are highly expressed in *Populus trichocarpa* and are differentially expressed in different tissues [32]. In wheat, natural variations in SuSy levels have been demonstrated to be explicitly associated with increased levels of cell wall polysaccharides [33]. SuSy enzyme activity is highly enriched beneath the secondary cell wall in differentiating tracheary elements during secondary wall formation in *Zinnea elegans* [34]. Similar results have been observed in wheat roots, which have increased cellulose content [13]. All these findings suggest that *SuSy* affect the sink strength by changing and/or converting carbohydrates, and it is also tightly coupled to cellulose biosynthesis.

*Populus simonii×Populus nigra* is a fast-growing and widely distributed tree species in Northern China, and it has been primarily used in the pulp and biofuel industries. However, knowledge about wood formation and the associated biological processes in this species is limited. We constructed two cDNA libraries from the cambial tissue of *Populus simonii* × *Populus nigra*. The results showed that *PsnSuSy2* is highly expressed in the xylem, similarly to the *SuSy2* in *Populus trichocarpa*. We thus postulated the *PsnSuSy2* is involved in wood formation in poplar xylem. In this study, we investigated the functions of *PsnSuSy2* by overexpressing this gene in tobacco, which has a secondary growth in stem that resembles that of poplar [35]. The tobacco transgenic lines were assessed for changes in transcript abundance, enzyme activity, biomass production, storage and structural polysaccharides, as well as cell wall ultrastructure. The results demonstrated that overexpression of *PsnSuSy2* channeled more carbon to cellulose and hemicellulose biosynthesis by at least partially sacrificing the carbon flow directed into lignin biosynthesis. These alterations resulted in changes in the fiber content, fiber length and the ultrastructural characteristics of the cell wall. The transgenic *PsnSuSy2* overexpression lines showed increased height, and no conspicuous deleterious effects on plant growth were observed.

## Materials and Methods

### Plant materials

Two-year-old *Populus simonii × Populus nigra* trees were propagated in the greenhouse owned by Northeast Forestry University (NEFU), Harbin, Heilongjiang Province, P.R. China. The use of the greenhouse was granted by NEFU. These poplar plants were then planted in a mixture of turfy peat and sand (2:1 v/v) in a greenhouse with 75% relative humidity and a temperature varying from 18°C ~ 28°C in May 2013. The cambium, phloem, xylem, old leaves, young leaves and roots were collected when plants were nearly one year old (one growth season) and then immediately frozen in liquid nitrogen and stored at -80°C for RNA isolation on August 15, 2013. The RNA was isolated according to a previously published method [36] and later treated with *DNase I* (Qiagen) to remove any remaining contaminating genomic DNA.

### Cloning the *PsnSuSy2* gene from *Populus simonii* × *Populus nigra*

*PsnSuSy* was cloned from two-year-old *Populus simonii × Populus nigra* trees. First, the first-strand cDNAs were synthesized from the RNAs isolated from the stem tissue of the hybrid trees by reverse transcription with the aid of the PrimeScript RT reagent Kit (Clontech.com). Secondly, we designed multiple pairs of primers based on the *SuSy2* gene of *Populus trichocarpa*. The full-length coding region of *PsnSuSy2* was obtained by high-fidelity PCR (Thermo Scientific) with two specific primers, *SuSy2*-F (5`-ATGTCTGTACTTACTCGTGTCC-3`) and *SuSy2*-R (5`-TTACTCGATAGTCAAAGGAACAG-3`) and cloned the PCR products into the pMD18-T cloning vector (TaKaRa Biotechnology, Dalian, China). This construct, pMD18*-PsnSuSy2*, was then transformed into *E*. *coli* cells (DH5α) for massive sequencing.

### Phylogenetic sequence analysis

A total of 116 putative plant SuSy2 protein sequences from both dicotyledon and monocotyledon plants were retrieved from GenBank at the National Center for Biotechnology Information (http://www.ncbi.nlm.nih.gov/genbank) and aligned with PsnSuSy2 using ClustalW within the MEGA 4.0 software package [37]. A phylogenetic tree was drawn with the same package using the neighbor-joining method with complete deletion; 1,000 replicates were used for bootstrap analysis, and the cutoff value was set to 50%.

### Plant transformation and maintenance

We cloned the *PsnSuSy2* into pROKII binary vector by double-digesting both pMD18*-PsnSuSy2*, and pROKII with BamHI 2μl (NEB) and KpnI (NEB) at the same time. The gel-purified *PsnSuSy2* and pROKII vector were ligated by T4 ligase (NEB). The obtained construct, pROKII*-35S-PsnSuSy2*, where *PsnSuSy2* is under the control of the 35S promoter, was transferred into *Agrobacterium tumefaciens* EHA105 using the freeze-thaw method. Transgenic tobacco plants were developed using a standard leaf disc inoculation method. First, *A*. *tumefaciens* containing EHA105 was incubated overnight in liquid MS medium supplemented with 3% sucrose and 100 μM acetosyringone. Then, the tobacco leaf discs were co-cultured with EHA105 for 1 h at room temperature, dried with a sterile paper towel, and placed abaxially onto MS medium supplemented with 0.1 μM each of α-naphthalene acetic acid (NAA) and 6-benzylaminopurine (BA). After three days, the discs were transferred onto selection medium of MS + NAA/BA containing carbenicillin disodium (500 mg/l) and cefotaxime sodium salt (250 mg/l) for three days. Then, the discs were transferred to MS + NAA/BA containing carbenicillin, cefotaxime and kanamycin (25 mg/l) and were cultured for approximately 4–5 weeks. Then, the shoot tips were cut and transferred into solid plain MS + 3%. The shoots of each transgenic line were cut and cultured until sufficient replicates were generated. Then, all plants were transferred to a greenhouse. The T1 seeds were collected from self-pollinated plants and were germinated on MS medium with kanamycin (25 mg/L) to produce T1 generation transgenic plants. Then, we repeated this procedure to obtain the T2 generation seeds, which were grown and used for all analyses conducted in this study. All plants were confirmed as transgenics by regular PCR screening of genomic DNA using gene-specific primers. All the transgenic and wildtype lines were grown in the greenhouse owned by Northeast Forestry University (NEFU), Harbin, Heilongjiang Province, P.R. China, and all the research activities were proven by NEFU.

### Biomass measurements

After three months of growth, the tobacco plants were harvested, and the total height and the stem diameter (3 cm above the root collar) were determined for each plant. The fresh weight was determined immediately. Then, the material was put into an oven and heated for ten minutes at 100°C. After that, the material was heated at 75°C until the weight did not change. This final weight was taken as the dry weight.

The plastichron index (PI) method was used to determine the stem growth. The first leaf with a length greater than 5 cm was defined as the first leaf and named PI0. Then, the leaf immediately below PI0 was defined as PI1. Stem segments between PI5 and PI8 were used in the wood cell wall, and chemical as well as puncture resistance analyses. Stem segments between PI3 and PI5 were retained for soluble carbohydrate content. Each of the above analyses was conducted with the five plants from each transgenic tobacco line and the wildtype.

### Determination of lodging resistance in transgenic tobacco

The puncture resistance of the stem segments between PI5 and PI8 from three-month-old transgenic lines and the wild type was analyzed using YYD-1 plant stalk analyzer according to the manufacturer’s instructions (Zhejiang Top Instrument Co., Ltd.).

### PCR, RT-PCR and real-time quantitative PCR analyses

The leaves of all transgenic lines were used to extract genomic DNA from one-month-old T2 transgenic plants using Depure Plant DNA kit (Deaou Biology Company, Guangzhou, China), and the genomic DNA from each line was used for PCR to verify the integration of *PsnSUSy2*. RT-PCR and real-time fluorescent quantitative PCR were used to determine the relative expression levels of *PsnSuSy2* in one-year-old clonal propagated *Populus simonii × Populus nigra* hybrid trees and transgenic tobacco lines. Five micrograms of total RNA from young leaf, old leaf, xylem, cambium, phloem and root tissue of the one-year-old poplar hybrids, as well as stem segments between PI3 and PI5 of wildtype and transgenic tobacco lines, were used for the synthesis cDNA using *SuperScript II* Reverse Transcriptase (Invitrogen, Carlsbad, CA, USA) and oligo (dT) 16 primers, according to the manufacturer’s instructions.

Samples were run in triplicate with the SYBR premix ExTaq kit (TaKaRa) and an Applied Biosystems 7500 Real-Time PCR System to determine the critical threshold (Ct). The primers used for RT-PCR analysis of *PsnSuSy2* were GS-RTF (5`-CGTGTCCAAAGCATTCGTGAACG-3`) and GS-RTR (5`-GCAATAATCTGGTGGTGTTGAAG-3`). *Actin*, a house-keeping gene, was used as a control for normalization. The primers used to quantify the *Actin* transcript were *Actin*-RTF (5`-AGGCAGGTTTCGCAGGAGATGA-`3) and *Actin*-RTR (5`-ACTTCCGGACATCTGAAC CT-3`) in poplar and *Actin*-RTF (5`-GATCTTGCTGGTCGTGATCT-3`) and *Actin*-RTR (5`-ACTTCCGGACATCTGAACCT-3`) in tobacco. The conditions for the RT-PCR reactions were as follows: 95°C for 10 min, followed by 40 cycles of 95°C for 30 seconds, 64°C for *PsnSuSy2* or 55°C for *Actin* for 1 min, and 72°C for 30 seconds. The relative expression was determined according to a previously described method [38] using the equation Δct = 2^-(ct*SuSy2*-ct*Actin*)^.

### Enzyme activity analysis

Stem samples (approximately 0.5 g) between PI3 and PI5 of two-month-old tobacco were ground in liquid nitrogen and then added 1 ml of extraction buffer that contained the following chemical compounds: 50mM N-2-hydroxyethylpiperazine-N′-2- ethanesulphonic acid (HEPES)-KOH, pH 7.5, 10 mM MgCl2, 1 mM ethylene diamine tetraacetic acid (EDTA), 2 mM dithiothreitol (DTT), 1 mM phenylmethylsulphonyl fluoride (PMSF), 5 mM ε-amino-n-caproic acid, 0.1% v/v Triton X-100, 10% v/v glycerol. The samples were centrifuged at 10,000 g for 10 min at 4°C. The extract was passed through a DG 10 desalting column and pre-equilibrated with ice-cold extraction buffer without Triton X-100. 50 μl plant extract was used for SuSy activity assay in the direction of sucrose breakdown as described [39]. This SuSy assay employed the controls without the supplementation of UDP to quantify inherent invertase activity, and thus the measured result reflected the breakdown of sucrose by SuSy. The resultant fructose content was determined by a tetrazolium blue assay [40].

### Chlorophyll measurement

The chlorophyll content was also measured with a CCM-200 instrument (http://www.optisci.com) using the third, fourth and fifth functional leaves from the two-month-old transgenic and the wildtype plants.

### Carbohydrate measurement

Approximately 50 mg of stem tissue was ground in liquid nitrogen. The samples were extracted with 1 ml of preheated 80% ethanol for 5 min at 80°C. Upon cooling, they were centrifuged at 12,000 g for 10 min. The supernatants were collected, and the pellets were resuspended in 0.5 ml of 50% ethanol and spun again as described above. The resulting pellet was extracted with 0.5 mL of water and re-centrifuged. The 2-ml total supernatant was mixed with an equal volume of chloroform and shaken vigorously. The aqueous phase was collected, dried in a vacuum, and redissolved in 0.5 ml water. The total soluble sugar, sucrose, glucose, and fructose contents were measured enzymatically on a spectrophotometer at 340 nm as described previously [41].

### Determination of the klason lignin content

The stem segments of three-month-old tobacco transgenic lines and the wildtype were ground to a powder after drying. The klason lignin content was analyzed using an ANKOM 2000i Automatic fiber analyzer.

### Fiber quality analysis

The stem segments with approximate dimension of 2 mm × 2 mm × 30 mm between PI5 and PI8 was harvested for analysis of the fiber quality. The harvested samples were immersed into Franklin solution (1:1 peroxide and glacial acetic acid) with 3.6% sodium hypochlorite for 20 h at 70°C. Upon decanting the solution, the materials were immersed in pure Franklin solution for 4 days at 70°C, and then washed in a vacuum with deionized water until the materials reached a neutral pH. The materials were dried for 24 h at 105°C and re-suspended in 10 ml of deionized water. The fiber length was obtained by counting 25–40 fibers per second on a Fiber Quality Analyser (FQA). All measurements were repeated three times.

### Scanning electron microscopy

The stem segments between PI5 and PI8 from L45 tobacco transgenic plants and the wildtype were used for scanning electron microscopy according to the method described by Carig and Beaton [42].

### Statistical analysis

Data on the *PsnSuSy2* height, stem diameter, fresh weight, dry weight, puncture resistance, expression levels, chlorophyll content, carbohydrate content, fiber quality, cellulose, hemicellulose and klason lignin content were analyzed using an unpaired *t*-test to assess the data generated from individual and pooled transgenic lines in comparison with non-transgenic controls. The significance level was set at *p* < 0.05.

### Ethics statement

The two-year-old *Populus simonii × Pululus nigra* trees were propagated in the greenhouse at Northeast Forestry University, Harbin, Heilongjiang province, P.R. China

## Results

### Cloning and phylogenetic tree analysis

Using primers derived from the *SuSy2* gene of *Populus trichocarpa*, a 2412-bp cDNA sequence was cloned by RT-PCR. This gene, hereafter, referred to as *PsnSuSy2*, encodes a protein with 385 amino acid residues, a molecular weight of 92.2 kDa and an isoelectric point of 6.0. The PsnSuSy2 amino acid sequence has 98% similarity to that of *Populus trichocarpa*, and analysis of the conserved domain of the protein suggested that the enzyme is a GT1 sucrose synthase.

**Fig 1:**
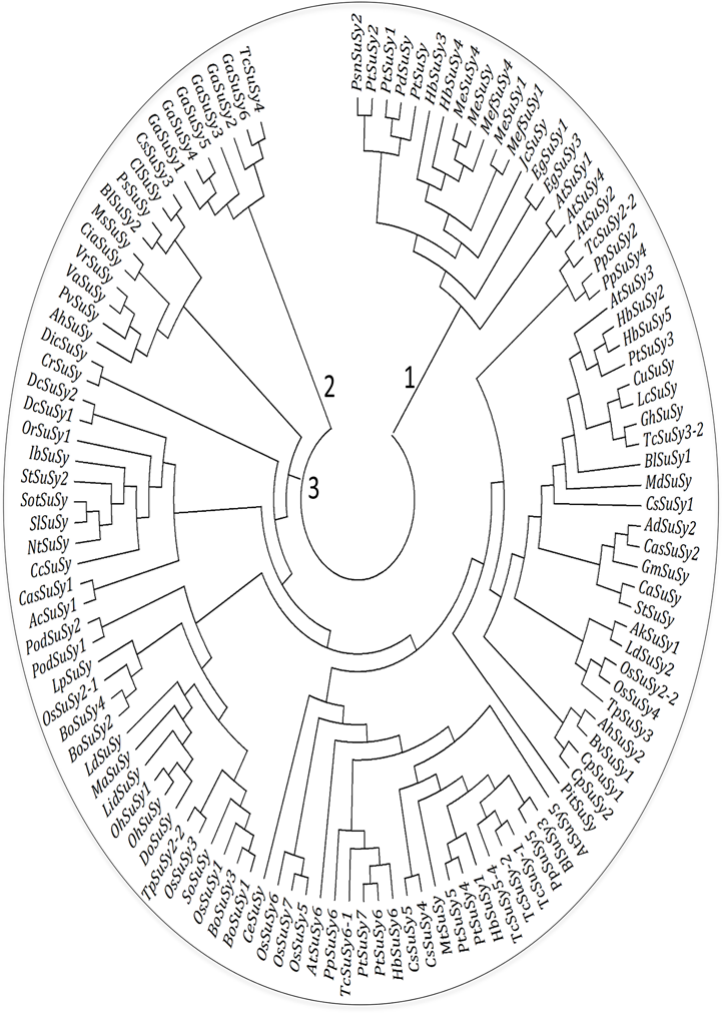
Phylogenetic tree of selected full SuSy coding sequences derived using the neighbor-joining method. The sequences were obtained by translating the following 116 full-length cDNA sequences of SuSy isozymes downloaded from Genbank (http://www.ncbi.nlm.nih.gov/genbank): *Arabidopsis thaliana* (AtSuSy1–6) isozyme1–6 (At5g20830, At5g 49190, At4g02280, At3g43190, At5g37180, At1g73370), *Actinidia chinensis* (AcSuSy) isozyme (AFO84090.1,), *Actinidia deliciosa* (AdSuSy) isozyme (AFO84091.1), *Amorphophallus konjac* (AkSuSy1) isozyme (AEH27530.1), *Arachis hypogaea* (AhSuSy2) isozyme (AFS17278.1), *Arachis hypogaea* var.vulgaris (AhSuSy) isozyme (AEF56625.1), *Bambusa oldhamii* (BoSuSy1–4) isozyme1–4(AAV64256.2, AAL50571.1, AAL50570.1, AAL50572.2), *Betula luminifera* (BlSuSy1–3) isozyme 1–3(AAV64256.2,AGV22112.1, AGV22113.1), *Beta vulgaris* (BvSuSy1) isozyme1 (ABR87939.1), *Camellia sinensi*s (CasSuSy1–2) isozyme1–2 (AHL29281.1, AHL29282.1), *Chenopodium rubrum* (*Cr*SuSy) isozyme (CAA57881.1), *Cicer arietinum* (*Cia*SuSy) isozyme (AEE60913.1,), *Citrus unshiu* (CuSuSy) isozyme(BAA88904.1), *Citrullus lanatus* (ClSuSy) isozyme (BAA89232.1), *Coccomyxa sub ellipsoidea* C-169(CeSuSy) isozyme (XP_005643464.1), *Coffea arabica* (CaSuSy) isozyme (CAJ32597.1), *Coffea canephora* (CcSuSy) isozyme (ABI17891.1), *Craterostigma plantagineum* (CpSuSy1–2) isozyme1–2 (CAB38021.1, CAB38022.1), *Cucumis sativus* (CsSuSy1,3–5) isozyme1,3–5 (AGA95977.1 AEN83999.1 AGA95976.1,P_001267613.1), *Daucus carota* (DcSuSy1,2) isozyme1,2 (CAA53081.1, CAA76057.1), *Dianthus caryophyllus* (DicSuSy) isozyme (BAJ10424.1), *Dendrobium officinale* (DoSuSy) isozyme (ADY02961.1), *Eucalyptus grandis* (EgSuSy1,3) isozyme1,3(ABB53601.1, ABB53602.1), *Gossypium arboreum* (GaSuSy1–6) isozyme1–6(AEV40460.1, AEV40461.1, AEV40462.1, AEV40463.1, AEV40464.1, AEV40465.1), *Gossypium hirsutum* (GhSuSy) isozyme (AIE38018.1), *Gunnera manicata* (GmSuSy)SuSy isozyme (ADP88918.1), *Hevea brasiliensis* (HbSuSy1–6) isozyme1–6(AGM14946.1, AGM14947.1, AGM14948, AGM14949.1, AGM14950.1, AGM14951. 1), *Ipomoea batatas* (IbSuSy) isozyme (ACL00957.1), *Jatropha curcas* (JcSuSy) isozyme (AGH29112.1), *Lilium davidii* (LidSuSy) isozyme (AGW23638.1), *Lilium davidii* var. unicolor(LdSuSy1,2) isozyme1,2(AHM02468.1, AHN50409.1), *Litchi chinensis* (LcSuSy) isozyme (AFP23359.1), *Lolium perenne* (LpSuSy) isozyme (BAE79815.1), *Manihot esculenta* (MeSuSy) isozyme (ABD96570.1), *Manihot esculenta* (MeSuSy1,4) isozyme1,4 (AIJ28962.1, AIJ28961.1), *Manihot esculenta subsp*. *Flabellifolia* (MefSuSy1,4) isozyme1,4 (AIJ28960.1,AIJ28959.1), *Malus domestica* (MdSuSy) isozyme (AFU56881.1), *Medicago sativa* (MsSuSy) isozyme (AAC17867.1), *Medicago truncatula* (MtSuSy) isozyme (XP_003616166.1), *Musa acuminata* (MaSuSy) isozyme (AEO09338.2), *Nicotiana tabacum* (NtSuSy) isozyme (AHL84158.1), *Oncidium hybrid* (OhSuSy, OhSuSy1) isozyme,1(AAM95943.1, AEA76429.1), *Orobanche ramose* (OrSuSy1) isozyme1 (AEN79500.1), *Oryza sativa*(OsSuSy1,3–7) isozyme1,3–7(|AEX32874.1, AEX32876.1, AEX32876.1, AEX32877.1, AEX32878.1, AEX32880.1), *Oryza sativa* (OsSuSy2–1, OsSuSy2–2) isozyme2–1,2–2 (AEX32875.1, AFI71274.1), *Populus simonii*×*Populus nigra* (PsnSuSy2) isozyme2, *Populus trichocarpa*(PtSuSy1–7) isozyme1–7(ADV71183.1, ADV71184.1, ADV71185.1, ADV71186.1, ADV71187.1, ADV71188.1, ADV71189.1), *Populus deltoids* (PdSuSy) isozyme (AHA41509.1), *Populus tremuloides* (PtSuSy) isozyme (AAR03498.1), *Potamogeton distinctus* (PodSuSy1–2) isozyme1–2(BAE06058.1, BAE06059.1), *Phaseolus vulgaris* (PvSuSy) isozyme (AAN76498.1), *Pinus taeda* (PitSuSy) isozyme (ABR15470.1), *Prunus serrulata* (PsSuSy) isozyme (AIL23782.1), *Prunus persica* SuSy (PpSuSy2,4–6) isozyme 2,4–6(AHZ90138.1, AHZ90140.1, AHZ90141.1, AHZ90142.1), *Prunus persica* (PpSuSy) isozyme(AHZ90138.1), *Solanum tuberosum* (SotSuSy) isozyme (AAA33841.1), *Solanum tuberosum* (StSuSy, StSuSy2) isozyme,2 (AAO67719.1, AAO34668.1), *Solanum lycopersicum* (SlSuSy) isozyme (AAA34196.1), *Saccharum officinarum* (SoSuSy) isozyme (AGI56230.1), *Theobroma cacao* (TcSuSy-1, TcSuSy-2) isozyme-1,-2 (XP_007032183.1, XP_007032184.1), *Theobroma cacao* (TcSuSy2–2) isozyme 2–2 (XP_007035652.1), *Theobroma cacao*(TcSuSy3–2) isozyme 3–2 (XP_007050985.1), *Theobroma cacao* (TcSuSy5–4) isozyme 5–4 (XP_007032186.1), *Theobroma cacao* (TcSuSy6–1) isozyme 6–1 (XP_007037101.1), *Triticum polonicum* (TpSuSy2–2) isozyme2–2 (AIL88517.1), *Triticum polonicum* (TpSuSy3) isozyme (AIL88515.1), *Vigna radiata* (VrSuSy) isozyme (BAA01108.1), *Vigna angularis* (VaSuSy) isozyme (BAH56282.1). The tree was generated using MEGA, version 4, with bootstrap of 1,000.

Phylogenetic analysis of PsnSuSy2 with 116 SuSy genes with full-length protein sequences from 55 species suggested the existence of three large phylogenetic classes (labeled as 1, 2 and 3 in Fig. 1). PsnSuSy2 was located in the first clade that contained 17 SuSy proteins from several woody tree/shrub species, which include but are not limited to *Populus trichocarpa* (PtSuSy1–2), *P*. *deltoids* (PdSuSy), *Hevea brasiliensis (*HbSuSy3, 4), *Eucalyptus grandis* (EgSuSy1,3), *Manihot esculenta* (MeSuSy1,4), and *Arabidopsis thaliana* (AtSuSy1 and 4). The second clade comprised seven proteins, six of them, GaSuSy1*~*6, were from *Gossypium arboretum* while one of them, TcSuSy4, was from *Theobroma cacao*. The third clade was the largest one, which comprised 93 SuSy proteins distributed in many large sub-clades (labeled from 4 to 15 in Fig. 1) at different levels. These 93 proteins belonged to 51 species that include grass, pine, rice, cocoa, wheat, peach, pondweed, tobacco, apple, and poplar etc. The genes from the same species were not necessarily located in the same sub-clades, and the proteins from both the monocotyledon and dicotyledon species could present in the same sub-clades of the second large clades, indicating the SuSy genes were evolved earlier.

### Tissue-specific expression analysis of *PsnSuSy2*

**Fig 2:**
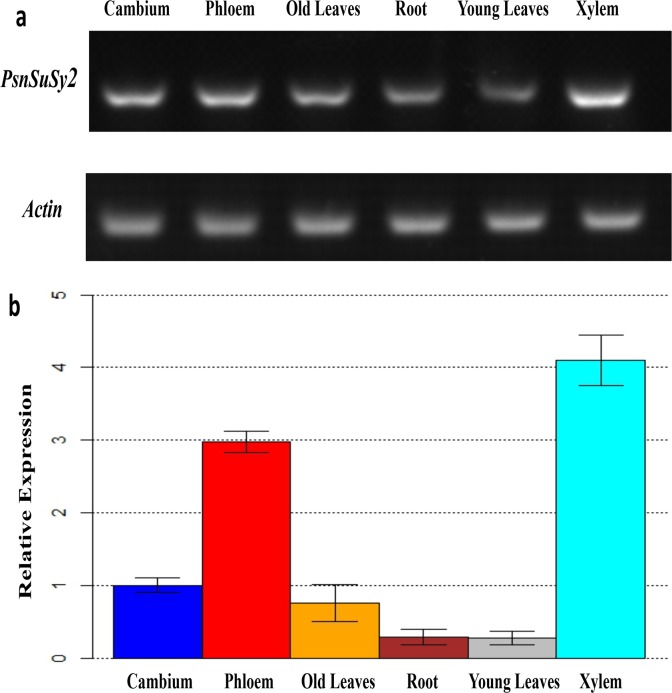
Tissue-specific expression of the *PsnSuSy2* in one-year-old hybrid *Populus simonii* × *Populus nigra* propagated from two years old hybrid trees. a. Tissue-specific expression pattern characterized by RT-PCR. b. Tissue-specific expression pattern characterized by real-time quantitative PCR. The expression levels for real-time quantitative PCR were averaged from three replicates.

Real-time RT-PCR was performed to investigate the *PsnSuSy2* gene expression patterns in various tissues of *Populus simonii* × *Populus nigra* using the gene-specific primers listed previously. The RT-PCR results demonstrated that the level of *PsnSuSy2* mRNA was the highest in the xylem compared to the other tissues (Fig. 2A). In addition, the expression levels in the phloem and cambium were also higher than those in the mature leaf and root. The lowest level of expression was found in young leaf tissue. The same result was obtained with Real-time RT-PCR (Fig. 2B). The expression levels of *PsnSuSy2* in the xylem and phloem were significantly higher than in the other tissues. The expression level of *PsnSuSy2* in the cambium was slightly higher than that in old leaves, but there was no significant difference between these two tissues. We also found that the expression level of *PsnSuSy2* in root was nearly the same as that in young leaves.

### Transgene tobacco expression analysis

**Fig 3:**
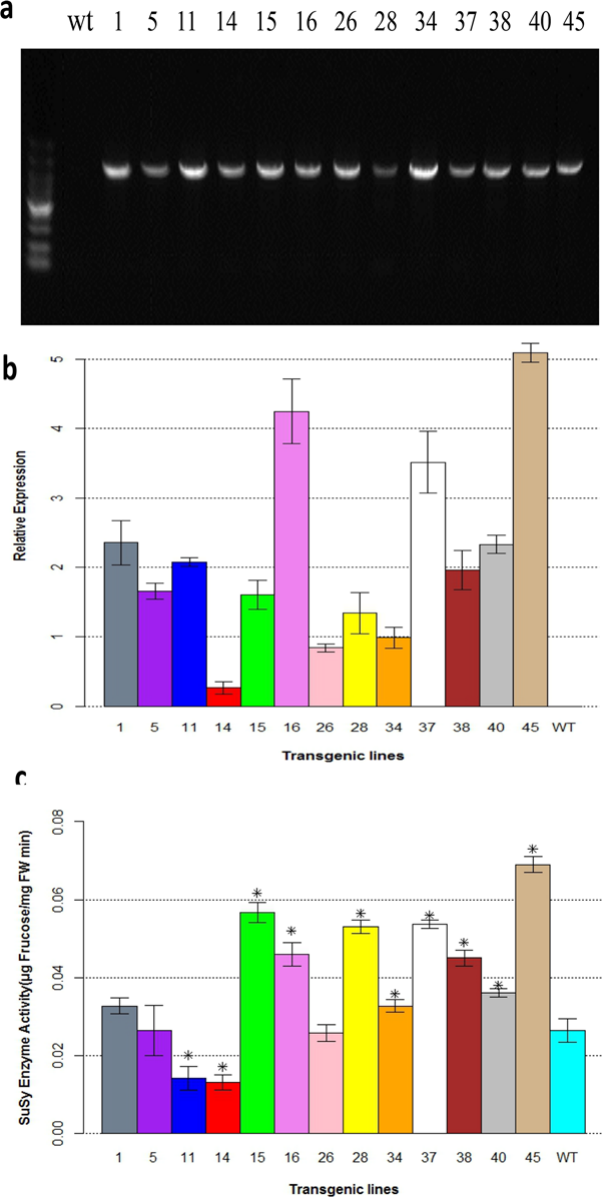
Validation of *PsnSuSy2* integration into tobacco genome and expression in transgenic tobacco lines. a. Validation of *PsnSuSy2* tobacco transgenic lines using PCR amplification. Genomic DNA was extracted from the leaves of one-month-old transgenic tobacco. The PCR products were electrophoresed on a 1.2% agarose gel with DL15000 DNA marker (first lane). b. Relative expression of *PsnSuSy2* in two month-old stem segments of plastichron index (PI) 3–5 determined by real-time RT-PCR. C. SuSy enzyme activity of in two month-old stem segments of PI3–5. Expressed levels in both Fig. 3b and 3c were averaged from five different samples per line ± S.E. The *t*-test was used to examine the significance of difference between *PsnSusy2* transgenic and wildtype lines, and *denotes significance at *p* < 0.05. WT represents wildtype tobacco while all others labeled with line numbers are of different *PsnSusy2* transgenic lines.

Thirteen T2 transgenic tobacco lines were verified by PCR using *PsnSuSy2* specific primers. The results indicated that *PsnSuSy2* was integrated into the tobacco genome (Fig. 3a). Real-time RT-PCR was then employed to characterize the *PsnSuSy2* expression levels relative to the housekeeping gene *Actin* in these verified transgenic tobacco lines (Fig. 3b). All lines exhibited varied expression levels of *PsnSuSy2*. Although the discrepancy in the expression levels was conspicuous, the *PsnSuSy2* expression levels in the L1, L11, L16, L37, L38, L40 and L45 transgenic lines were relatively higher than in the other transgenic and the wildtype (Fig. 3b). To examine whether *PsnSuSy2* was correctly translated into a functional protein in the transgenic lines, the SuSy activity was examined by measuring the breakdown of the sucrose cleavage products, fructose. The results were notably different in the stems of the transgenic lines and the wildtype (Fig. 3c). The activity of SuSy in the nine transgenic lines was higher than that in the wildtype,, ranging from 0.033 to 0.069 μg fructose /mg FW min in the transgenic lines compared with 0.026 μg fructose /mg FW min in the wildtype, representing a 2-fold increase in the enzymatic activity in the transgenic lines. Although a couple of lines, for example L15 and L28, did not exhibit consistent *PsnSuSy2* mRNA expression levels and PsnSuSy enzyme activity in Fig. 3b and 3c, most other transgenic lines were indeed consistent, indicating the mRNAs of *PsnSuSy2* could generally be translated into the protein efficiently in transgenic lines.

### Changes in growth and morphology

**Fig 4:**
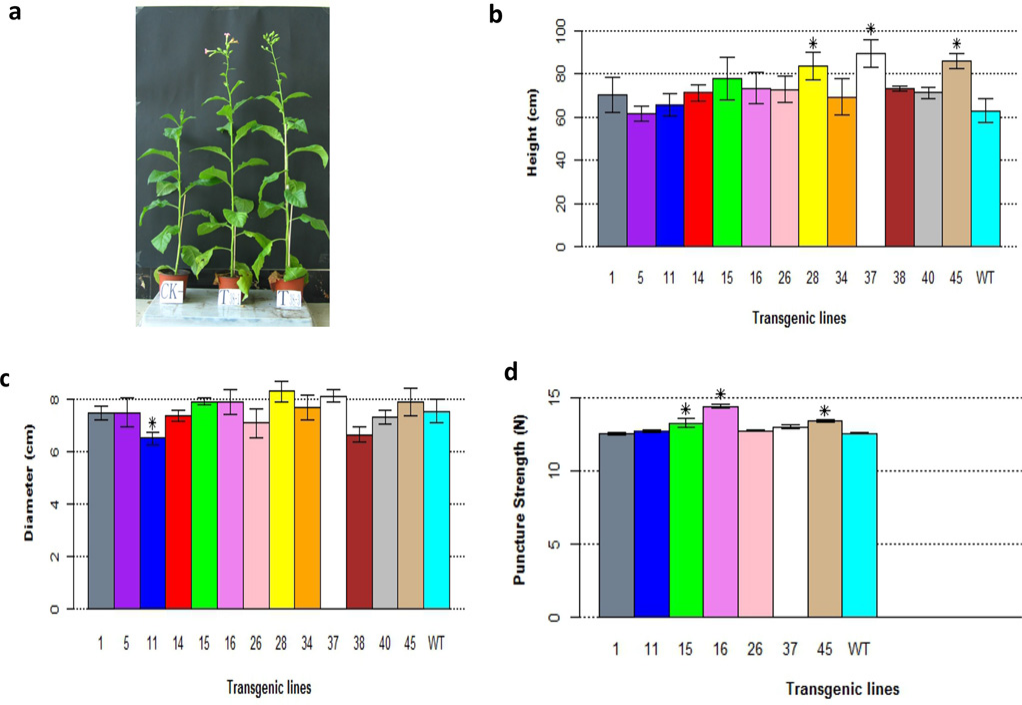
Phenotypic changes in *PsnSusy2* transgenic lines. a. Phenotypic comparison of three-month-old tobacco transgenic line of L38 and wildtype tobacco. CK represents wildtype tobacco while T represents L38 transgenic line. b. Comparison of heights of three-month-old tobacco transgenic lines and wildtype. c. Stem diameters of three-month-old tobacco transgenic lines at 3 cm height above the root collar. d. Puncture strength of stem segments (plastichron index 5) in three-month-old transgenic lines. All measurements shown in a, b, and c were averaged from five different samples per line ± S.E. The *t*-test was used to examine the significance of difference between *PsnSusy2* transgenic and wildtype lines, and *denotes significance at *p* < 0.05. The WT represents wildtype tobacco while the others lines labeled with line numbers are of different *PsnSusy2* tobacco transgenic lines.

Compared to the wildtype, the transgenic lines of *PsnSuSy2* grew faster throughout their lives, which led to a difference in the plant heights and stem diameters (Fig. 4A). The differences in the heights of transgenic lines were more obvious than the differences in the stem diameters, and 12 out of 13 transgenic lines were taller than the wildtype. Of these 12 lines, L28, L37 and L45 were significantly taller than the wildtype (Fig. 4B). The difference in stem diameter was subtle. With the exception of L11, which had significantly smaller stem diameter than the wildtype, no significant differences were found in stem diameters between the transgenic lines and the wildtype (Fig. 4C). The stem stiffness of seven transgenic lines measured by puncture was consistently higher than that of the wildtype (Fig. 4D). Of these seven lines, the stiffness of the transgenic lines L15, L16 and L45 was significantly higher than that of the wildtype.

**Fig 5:**
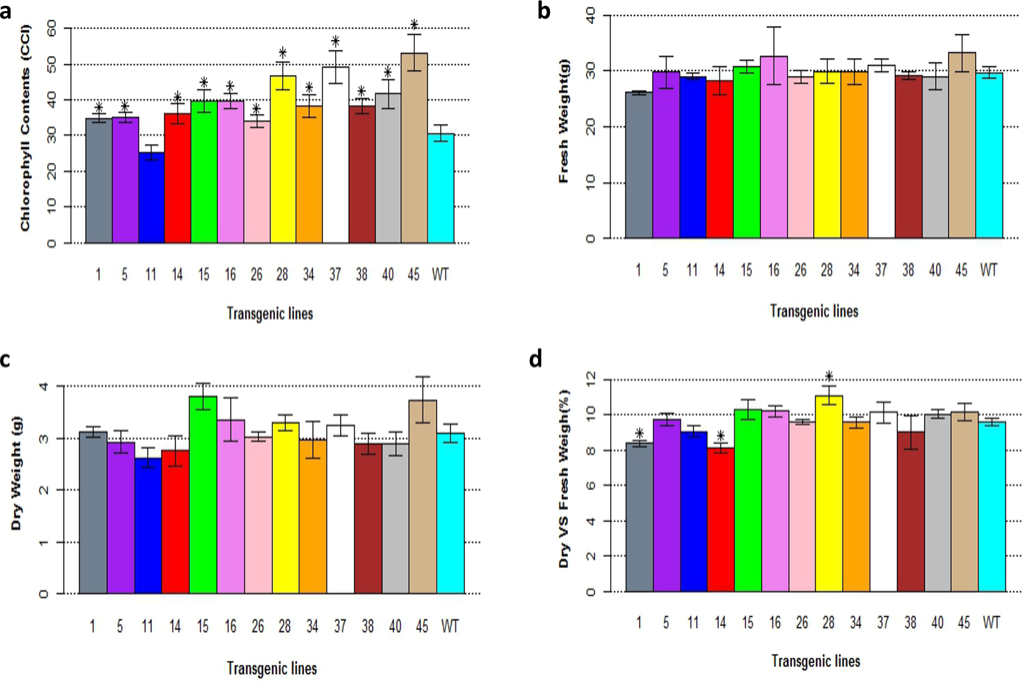
Changes in chlorophyll contents and biomass of *PsnSusy2* tobacco transgenics. a. Chlorophyll contents of the third, fourth and fifth functional leaves of different *PsnSuSy2* tobacco transgenic lines of two-month-old plants. b. Fresh weights of three-month-old *PsnSuSy2* tobacco transgenic lines. c. Dry weights of three-month-old *PsnSuSy2* tobacco transgenic lines. d. Ratios of dry vs. fresh weight for of three-month-old *PsnSuSy2* tobacco transgenic lines. All measurements shown in 5a, 5b, and 5c were averaged from five different samples per line ± S.E. The *t*-test was used to examine the significance of difference between *PsnSusy2* transgenic and wildtype lines, and *denotes significance at *p* < 0.05. The WT represents wildtype tobacco while the others lines labeled with line numbers are of different *PsnSusy2* tobacco transgenic lines.

We also found that the leaves of the transgenic lines were greener compared to those of the wildtype plants, which might imply that the chlorophyll contents in the *PsnSuSy2* transgenic lines were higher. We found that the chlorophyll contents in all transgenic lines except L11 were significantly higher than that of the wildtype (Fig. 5A). The changes in the chlorophyll contents might increase photosynthesis, which could potentially increase the biomass of the transgenic tobacco plants. However, as shown in Fig. 5B-D, the biomass of the transgenic lines was rarely significantly different from that of the wildtype. The fresh weights of L16 and L45 and the dry weights of L15 and L45 appeared to be much higher than that of the wildtype. However, the differences were not significant. Nevertheless, the ratio of dry weight to fresh weight in L28 was significantly higher than that of the wildtype, and on the contrary, the ratios of dry weights to fresh weights in L1 and L14 were significantly lower than that of the wildtype.

### Soluble carbohydrates and sugars

**Fig 6:**
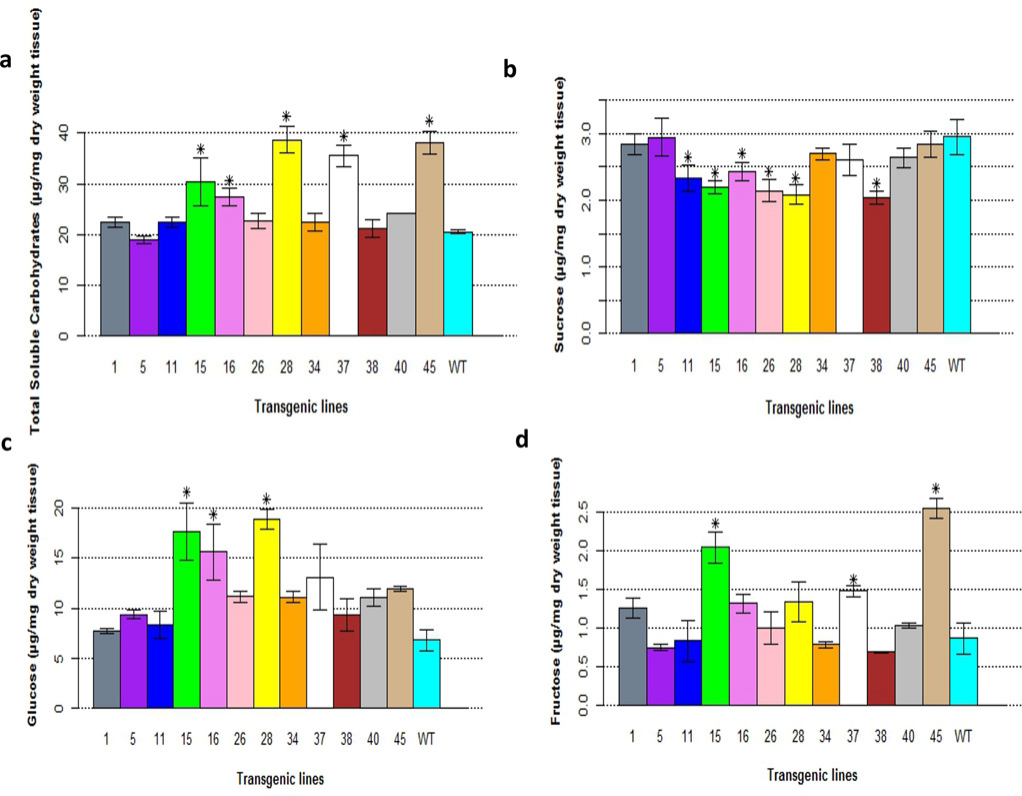
Changes in carbohydrates (sugars) in *PsnSusy2* tobacco transgenics. a. Total soluble sugar contents of stem tissues in different *PsnSuSy2* tobacco transgenic lines. b. Sucrose contents of stem tissues in different *PsnSuSy2* tobacco transgenic lines. c. Glucose contents of stem tissues in different *PsnSuSy2* tobacco transgenic lines. d. Fructose contents of stem tissue in different *PsnSuSy2* tobacco transgenic lines. All samples in 6a, 6b and 6c were harvested from tissues between PI3 and PI5. All measurements shown in 6a-d were averaged from five different samples per line ± S.E. The *t*-test was used to examine the significance of difference between *PsnSusy2* transgenic and wildtype lines, and *denotes significance at *p* < 0.05. The WT represents wildtype tobacco while the others lines labeled with line numbers are of different *PsnSusy2* tobacco transgenic lines. Note that the total soluble sugar contents were measured using two-month-old plants, whereas sucrose, glucose, and fructose were measured one week later.

The SuSy enzymes play an important role in plant sugar metabolism, and the overexpression of *PsnSuSy2* in transgenic lines could affect the soluble sugar content. As shown in Fig. 6A, the levels of total soluble carbohydrate increased in all transgenic lines except L5, although the increase in total soluble carbohydrate only reached significance in five lines, L15, 16, L28, L37, and L46. As anticipated, all 13 *PsnSuSy2* transgenic tobacco lines had lower levels of sucrose in the stem segments, and six of these 13 lines had statistically significantly lower levels of sucrose compared to the wildtype (Fig. 6B). On the contrary, all 13 transgenic lines had consistently higher glucose contents, though only three of them were beyond the level of significance (Fig. 6C). Eight lines had higher fructose levels, and this difference was statistically significant in three of these (Fig. 6D).

### Changes in the thickness and composition of the secondary cell wall

**Fig 7:**
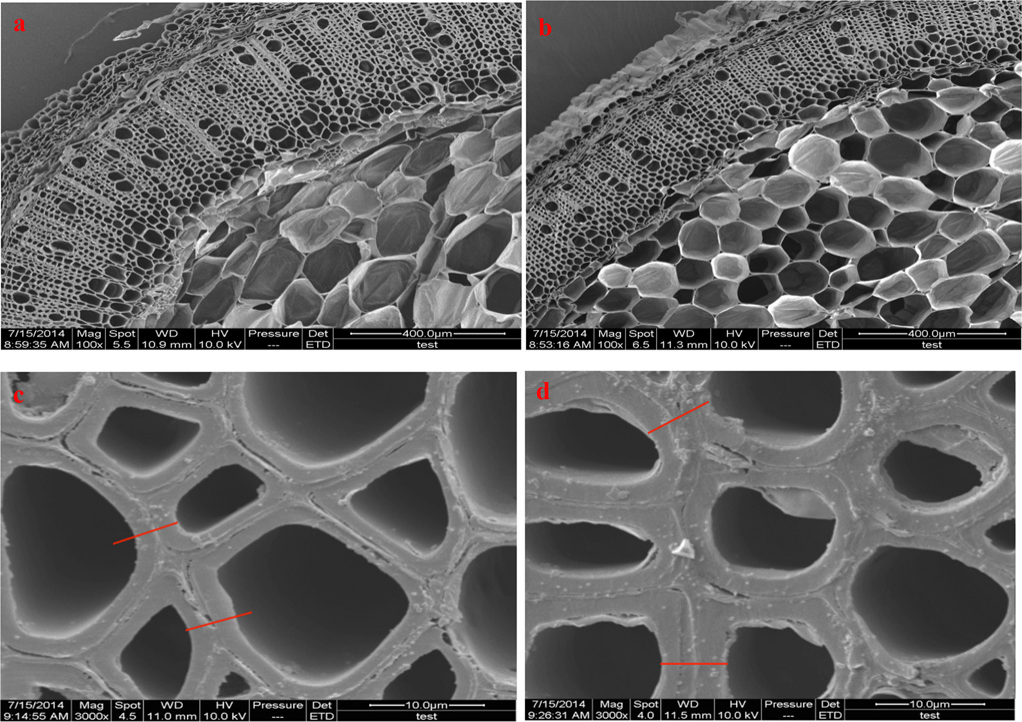
Scanning electron micrographs of the PI5–8 stem transverse sections of three-month-old tobacco transgenic lines. a. and c were from wildtype tobacco plants. b. and d are the L15 *PsnSusy2* tobacco transgenic line. Short red lines in Figure c and d depict the difference between the cell wall thickness in the tobacco transgenic lines and the wildtype plants. The magnification factor is 100X for Fig. 7a and 7b, and 3000X for 7c and 7d.

**Fig 8:**
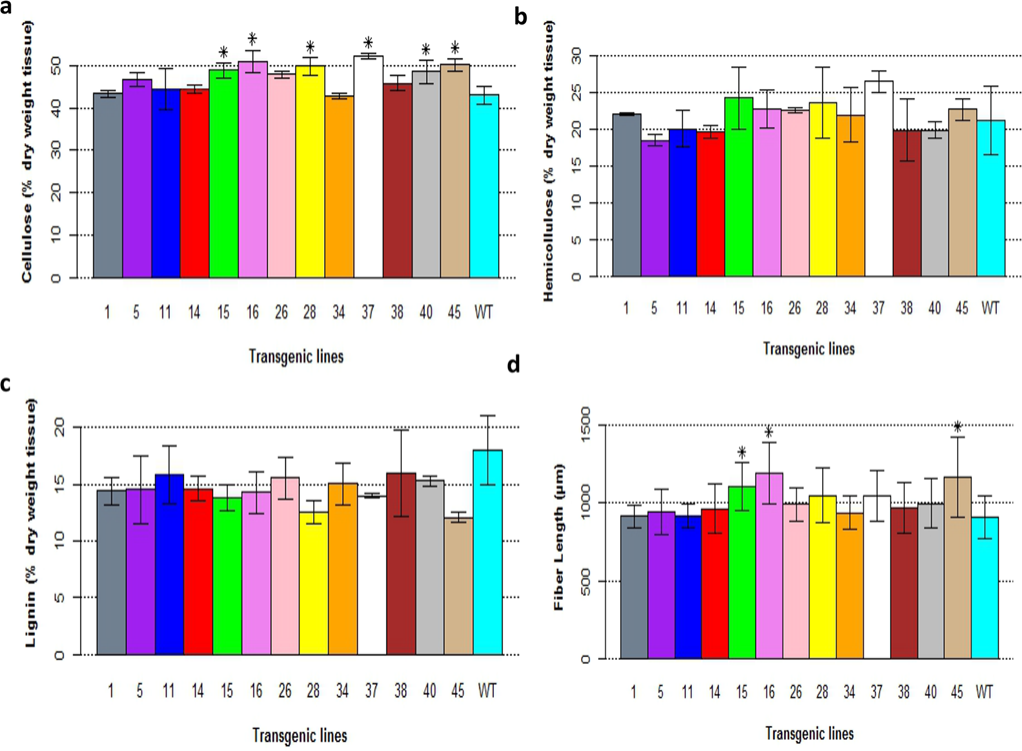
Changes in lignocellulosic components in *PsnSusy2* tobacco transgenic lines. a. Cellulose contents in the plastichron index (PI) 5~8 stem tissues of three-month-old *PsnSusy2* transgenic tobacco lines. b. Hemicellulose contents in the PI5~8 stem tissues of three-month-old *PsnSusy2* transgenic tobacco lines. c. Lignin contents in the stem the PI5~8 stem tissues of three-month-old *PsnSusy2* transgenic tobacco lines. d. Fiber length in the PI5~8 stem tissue of three-month-old *PsnSusy2* transgenic tobacco lines. All measurements are the means of five different samples ± S.E. The *t*-test was used to examine the significance of difference between *PsnSusy2* transgenic and wildtype lines, and *denotes significance at *p* < 0.05. The WT represents wildtype tobacco plants, whereas all the others are different *PsnSusy2* transgenic lines.

Scanning electron micrographs (SEM) clearly showed a thickened secondary cell wall in the L15 transgenic line when compared with the wildtype (Fig. 7A-D). The cell wall in the L15 line was estimated to be 25% thicker compared to that of the controls. To identify which components (e.g., cellulose, lignin, or hemicellulose) gave rise to the increased thickness of the secondary cell wall, we analyzed the composition of cell wall using an ANKOM 2000i Automatic Fiber Analyzer. All transgenic lines except L34 had higher cellulose contents than the wildtype (Fig. 8A). Six of the transgenic lines had significantly higher cellulose contents (L15, L16, L28, L37, L40, and L45) (Fig. 8A). The hemicellulose content increased in eight lines, but decreased in five lines though none of these differences reached the level of significance (Fig. 8B). In contrast to cellulose, we found that the lignin content of all transgenic lines was decreased. However, there was no significant difference in lignin content between any of the transgenic lines and the wildtype (Fig. 8C). These results implied that the higher cellulose content was responsible for the thickened secondary cell wall. Finally, we investigated whether the increased cellulose biosynthesis had any effect on the fiber length (Fig. 8D). Surprisingly, the fiber lengths in all transgenic lines were longer than in the wildtype, and the fiber lengths in three transgenic lines, L15, L16, and L45, increased significantly (Fig. 8D).

## Discussion

To explore the evolutionary relationship between *PsnSuSy2* and other *SuSy* genes in other plant species, a rooted neighbor-joining phylogenetic tree was constructed using 116 full-length SuSy protein sequences available in Genbank (http://www.ncbi.nlm.nih.gov/genbank). The plant SuSy proteins could be subdivided into three clearly distinguished clades with high statistical confidence (Fig. 1). Clade 1 included PsnSuSy2 and 13 other SuSy proteins from the tree and shrub species that included *Populus trichocarpa* (PtSuSy1–2), *P*. *deltoids* (PdSuSy), *Eucalyptus grandis* (EgSuSy1,3), *Manihot esculenta* (MeSuSy), *Manihot esculenta*(MeSuSy1,4), *Manihot esculenta subsp*. *Flabellifolia* (MefSuSy1,4). In addition, there was one gene from *Jatropha curcas* (JcSuSy) and two from *Arabidopsis* (AtSuSy1, and 4) in Clade 1. It is not surprising that SuSy proteins of Poplar and *Arabidopsis* showed up in the same clade because genome studies suggested poplar and *Arabidopsis* recently diverged (~110 Myr ago) and are known to be closely related [43–45]. The coexistence of PsnSuSy2 with several SuSy proteins from other tree species in Clade 1 indicated the SuSy proteins in tree species have evolved and become different from others. The presence of SuSy proteins from the monocotyledon, and dicotyledon species as well as the gymnosperms species in the Clade 3 suggests that the *SuSy* genes were evolved before the divergence of monocotyledon and dicotyledon species (~140~200 Myr ago) [46, 47] and also earlier than the divergence of angiosperms and gymnosperms (~300 Myr ago) [43], which is evolutionarily plausible because cell wall biosynthesis was indispensable when plants were first emerged. Taken together, we suggest that *PsnSuSy2* plays a key role in cleaving sucrose for cellulose biosynthesis in stem tissue.

The function of *PsnSuSy2* was investigated by overexpressing this gene in tobacco plants, which have a stem structure that resembles poplar [35] and can be used to characterize genes that may be involved in wood formation and the associated biological processes. In the *PsnSuSy2* transgenic tobacco lines, the SuSy activity in the developing stems was significantly increased, which resulted in decreased sucrose levels in most lines, increased glucose levels in all lines, and increased fructose levels in most lines, indicating the poplar PsnSuSy2 cleaved more sucrose into UDP-glucose and fructose monomers in tobacco. This result is in agreement with a previous study that demonstrated that the overexpression of the *Gossypium hirsutum SuSy* gene in tobacco led to reduced sucrose contents and elevated glucose and fructose levels [27]. We also found that the cellulose and hemicellulose contents of some transgenic lines increased significantly, whereas the acid-soluble lignin contents in same tobacco transgenic lines decreased slightly (Fig. 8C), and a negative correlation was found between cellulose and lignin contents (correlation coefficient = - 0.62). In several other plant species, the expression of *SuSy* has been shown to affect cellulose synthesis. For example, over-expression of a mung bean *SuSy* enhances the production of cellulose biosynthesis in *Acetobacter xylinum*, a bacterium where there is no SuSy and the synthesis of UDP-glucose from sucrose takes more than four steps. The mung bean sucrose synthase not only serves to channel carbon directly from sucrose to cellulose biosynthesis in bacteria but also promote UDP recycle soon after UDP formed in synthetic direction [48]. The accumulation of UDP is known to inhibit cellulose biosynthesis through a feedback loop [48]. SuSy is also proposed to facilitate cellulose biosynthesis by channeling UDP-glucose to cellulose synthase [16]. By contrast, down-regulation of *SuSy* in carrot reduced the cellulose content [49], and suppression of *SuSy* in cotton resulted in an almost fiberless phenotype [25]. Additionally, transgenic lines of aspen with strongly reduced SuSy activity in developing wood were reported to have no impact on phenotype but the contents of lignin, hemicellulose and cellulose were decreased significantly per wood volume [50]. In addition to generating UDP-glucose for accelerating cellulose biosynthesis, several studies suggest that at least some sucrose synthases are present in plasma membrane-bound forms [34, 51, 52] and participate in providing carbon for cellulose biosynthesis. Fujii et al. [17] suggested that SuSy serves as a part of a large soluble catalytic domain of the CesA complex. Another interesting finding in our study is that the overexpression of *PsnSuSy2* resulted in a thickened cell wall in tobacco (Fig. 7d). The thickened cell wall contained up to 18% more cellulose (L37) and 28% less lignin (L45) compared to the wildtype (Fig. 8a,b), suggesting that the overexpression of *PsnSuSy2* resulted in the channeling of more carbon for cellulose biosynthesis. This conclusion is also supported by the finding that *SuSy2* was preferentially expressed in the stem xylem (Fig. 2) where it was coupled to cellulose biosynthesis. A previous study in cotton indicated that more than 50% of SuSy is associated with the plasma membrane and functions to degrade sucrose for the synthesis of cell wall polysaccharides and starch [53].

Surprisingly, the *PsnSuSy2*-overexpressing transgenic tobacco lines did not show any severe developmental defects. Rather, overexpression of the *PsnSuSy2* had a noticeable effect on tobacco growth. We found that most of the transgenic lines were taller than the controls, but no significant increase in the stem diameters was observed. Though the biomass in some of transgenic lines, such as L15 L45, and L28, was obviously higher than that of the controls (Fig. 4b), there was no significant difference in biomass between most of the transgenic and wildtype lines (Fig. 5). This is in agreement with the conclusion of Coleman et al. that the overexpression of *Gossypium hirsutum* sucrose synthase (*SuSy*) in tobacco resulted in increased height and longer internode length [27]. Xu et al reported that overexpression of potato *SuSy* accelerates leaf expansion and plant height growth [28]. Xu and Joshi found that overexpression of the aspen sucrose synthase gene in *Arabidopsis* promotes the growth and development of transgenic *Arabidopsis* plants [54]. In addition, a modified version of *SuSy* driven by the 35S promoter from *Vigna radiate* was reported to increase the plant height in some transgenic lines of *Populus alba* [55]. On the contrary, the suppression of *SuSy* has been demonstrated to reduce plant height in several species. For example, using antisense-mediated suppression, Baier et al. [56] showed that down-regulation of a *SuSy* leads to decreased height in *Medicago truncatula*. D'Aoust et al. [57] produced tomatoes with a small size due to the reduced capacity to transfer sucrose to the fruits. Similar results have also been reported in other studies [15, 27, 58, 59]. Because the depletion of sucrose through the cleavage activity of SuSy generally affects sink strength, it is not surprising that there is a correlation between increased organ size and SuSy activity, whereas down-regulation of SuSy activity can result in decreased growth.

We observed that the chlorophyll contents of all transgenic tobacco lines except one were significantly increased compared to wildtype. The increase in chlorophyll contents would presumably lead to augmented photosynthesis. However, there was no significant gain in biomass in most of the transgenic lines (Fig. 5). The relationship between the chlorophyll content and the overall biomass (fresh and dry weight) in the different lines was unclear. However, a correlation between the chlorophyll content and plant height (Figs. 5a and 4b respectively) was conspicuous. Currently, it is unclear why the *PsnSuSy2* transgenic tobacco plants had high chlorophyll content, but this observation was certainly related to the depletion of the product of photosynthesis, sucrose, and the augmented sink strength resulting from *PsnSuSy2* overexpression.
